# Ibrutinib selectively and irreversibly targets EGFR (L858R, Del19) mutant but is moderately resistant to EGFR (T790M) mutant NSCLC Cells

**DOI:** 10.18632/oncotarget.5182

**Published:** 2015-09-05

**Authors:** Hong Wu, Aoli Wang, Wei Zhang, Beilei Wang, Cheng Chen, Wenchao Wang, Chen Hu, Zi Ye, Zheng Zhao, Li Wang, Xixiang Li, Kailin Yu, Juan Liu, Jiaxin Wu, Xiao-E Yan, Peng Zhao, Jinhua Wang, Chu Wang, Ellen L. Weisberg, Nathanael S. Gray, Cai-Hong Yun, Jing Liu, Liang Chen, Qingsong Liu

**Affiliations:** ^1^ High Magnetic Field Laboratory, Chinese Academy of Sciences, Hefei 230031, Anhui, P. R. China; ^2^ University of Science and Technology of China, Hefei 230036, Anhui, P. R. China; ^3^ Collaborative Innovation Center of Cancer Medicine, National Institute of Biological Sciences, Beijing, Beijing 102206, P.R. China; ^4^ Synthetic and Functional Biomolecules Center, Beijing National Laboratory for Molecular Sciences, Key Laboratory of Bioorganic Chemistry and Molecular Engineering of Ministry of Education, College of Chemistry and Molecular Engineering, Peking-Tsinghua Center for Life Sciences, Peking University, Beijing 100871, P.R. China; ^5^ Institute of Systems Biomedicine, Department of Biophysics, Beijing Key Laboratory of Tumor Systems Biology and Center for Molecular and Translational Medicine, School of Basic Medical Sciences, Peking University Health Science Center, Beijing 100191, P.R. China; ^6^ Department of Cancer Biology, Dana-Farber Cancer Institute, and Department of Biological Chemistry & Molecular Pharmacology, Harvard Medical School, Boston, MA 02115, USA; ^7^ Department of Medical Oncology, Dana Farber Cancer Institute, Harvard Medical School, Boston, MA 02115, USA; ^8^ Hefei Science Center, Chinese Academy of Sciences, Hefei 230031, Anhui, P. R. China

**Keywords:** ibrutinib, NSCLC, EGFR mutation, drug resistance, drug combination

## Abstract

Through comprehensive comparison study, we found that ibrutinib, a clinically approved covalent BTK kinase inhibitor, was highly active against EGFR (L858R, del19) mutant driven NSCLC cells, but moderately active to the T790M ‘gatekeeper’ mutant cells and not active to wild-type EGFR NSCLC cells. Ibrutinib strongly affected EGFR mediated signaling pathways and induced apoptosis and cell cycle arrest (G0/G1) in mutant EGFR but not wt EGFR cells. However, ibrutinib only slowed down tumor progression in PC-9 and H1975 xenograft models. MEK kinase inhibitor, GSK1120212, could potentiate ibrutinib's effect against the EGFR (L858R/T790M) mutation *in vitro* but not *in vivo*. These results suggest that special drug administration might be required to achieve best clinical response in the ongoing phase I/II clinical trial with ibrutinib for NSCLC.

## INTRODUCTION

Ibrutinib (PCI-32765), an irreversible BTK kinase inhibitor, has been extensively studied in a variety of hematopoietic malignancies, such as mantle cell lymphoma (MCL), chronic lymphatic lymphoma (CLL), diffuse large B-cell lymphoma (DLBCL), multiple myeloma (MM), and acute myeloid leukemia (AML). Recently it was approved for the clinical treatment of MCL and CLL. [1–7] Ibrutinib has also been studied in preclinical inflammation models. [8–10] Besides its potent activity against BTK kinase, previous studies have shown that ibrutinib may also potently inhibit BMX, BLK, EGFR, and Her2 kianses. [10]

EGFR is a receptor tyrosine kinase that regulates downstream signaling cascades such as PI3K/Akt/mTOR and Ras/RAF/MEK/ERK etc. [11] Gain-of-function mutations of EGFR such as L858R, exon 19 deletion (Del 19), which account for around 90% of all EGFR mutations, can lead to tumorigenesis. [12] First generation EGFR inhibitors, such as Gefitinib and Erlotinib, have exhibited great anti-tumor activity against the EGFR (L858R) and EGFR (del 19) mutation- driven NSCLC. [13] However, the efficacy of chronic treatment with Gefitinib and Erlotinib is at best transient due to signaling bypass resistance mechanisms such as Her2 and MET amplification, or acquired EGFR gatekeeper T790M mutation. [14, 15] This has encouraged the development of second-generation EGFR inhibitors such as Apatinib (BIBW2992), Neratinib and Dacomitinib, which work through formation of a covalent bond with Cys797. [16–18] However, due to the lack of selectivity between the wt EGFR and mutant EGFR, these drugs showed dose-limiting toxicities, which led to develop third generation EGFR (T790M) inhibitors, such as WZ4002, CO-1686 and AZD9291. [19–22]

Recently ibrutinib has been reported to exhibit anti-tumor activities in EGFR mutant NSCLC and currently under phase I/II clinical trial for the previously treated NSCLC (NCT02321540). [23] Combining the previously reported biochemical activity of ibrutinib against wt EGFR and the fact that similar reactive cysteine residues are located at the same positions in EGFR and BTK, we performed a comprehensive comparison study of ibrutinib against NSCLC *in vitro* and *in vivo*. We found that ibrutinib exhibited selective anti-proliferation activity against EGFR primary mutants (L858R, del19)-expressing cancer cells but moderately active against drug induced secondary gate-keeper T790M mutation and not active against wt EGFR-expressing cells. [10] In addition, ibrutinib exhibited superior selectivity between mutant and wide type EGFR in comparison with clinical stage EGFR inhibitors such as BIBW2992, CO1686 and AZD9291. Ibrutinib could be effectively combined with MEK kinase inhibitor GSK1120212 against the EGFR secondary mutant (L858/T790M). However, these potent anti-proliferation activities could not directly transform into PC-9 and H1975 xenografts models. These results indicated that ibrutinib might be a potential drug candidate for the EGFR (L858R, del19) mutant driven NSCLC and a useful candidate for combinatorial therapy aimed at overcoming (L858R/T790M) resistance in NSCLC. But considering the discrepancies observed between the *in vitro* and *in vivo*, special administration design might be required to achieve the best drug response in the solid NSCLC tumor.

## RESULTS

### Ibrutinib selectively inhibits the proliferation of EGFR mutant NSCLC cancer cell lines

We first screened a panel of NSCLC cancer cell lines and found that only mutant EGFR-expressing cell lines, such as H3255 (EGFR L858R, GI_50_: 0.11 μM), PC-9 (EGFR Del 19, GI_50_: 0.05 μM), and HCC827 (EGFR Del 19, GI_50_: 0.063 μM), are sensitive to ibrutinib treatment. (Table 1) A similar trend was observed with other EGFR inhibitors, including BIBW2992, WZ4002, CO-1686, AZD9291 and Gefitinib. Interestingly, the H1975 cell line (EGFR L858R/T790M) was only moderately sensitive to ibrutinib (GI_50_: 1.2 μM) compared with other EGFR T790M mutation-targeting drugs. Unlike the other agents, ibrutinib did not exhibit any inhibitory effect against wt EGFR-expressing cancer cell lines, which suggests a superior selectivity window (Table 1). Intriguingly, a reversible version of ibrutinib (PCI-R, structure shown in Fig. S1), in which the acrylamide is replaced with a propionamide group, lost most of the activity against these otherwise sensitive cell lines. To further confirm the activity of ibrutinib against mutant EGFR-expressing cell lines, we conducted colony formation assays. The results demonstrated that for the EGFR L858R/T790M mutant-driven H1975 cell line, ibrutinib showed moderate anti-colony formation activity (EC_50_: 604.7 nM), which is similar to that of CO-1686 (EC_50_: 605.8 nM). (Table S1 and Fig. S2A) WZ4002 showed the strongest inhibitory activity (EC_50_: 12 nM) and AZD9291 exhibited less activity (EC_50_: 118 nM). AZD9291 exhibited an EC_50_ of 2.5 nM against the PC-9 cell line. Ibrutinib and WZ4002 showed similar efficacy (EC_50_: 16 nM and 11 nM respectively), while CO-1686 exhibited an EC_50_ of 56 nM. (Fig. S2B) AZD9291 demonstrated the strongest activity against the HCC827 cell line (EC_50_: 1.2 nM). WZ4002 and CO-1686 showed similar activities (EC_50_: 10 nM and 11 nM respectively), while ibrutinib was relatively less potent (EC_50_: 155 nM) (Fig. S2C) For H3255 cell line, AZD9291 displayed the strongest anti-colony formation activity (EC_50_: 5.5 nM). Ibrutinib and WZ4002 were moderately active (EC_50_: 23.4 nM and 89 nM), whereas CO1686 was relatively less active (EC_50_: 210.8 nM). (Fig. S2D)

**Table 1:** ibrutinib anti-proliferation efficacy against EGFR wt/mutant NSCLC cell lines

Cell LineGI_50_(μM)	EGFR status	BIBW-2992	WZ-4002	ibrutinib	PCI-R	CO-1686	AZD-9291	Gefitinib
H3255	EGFRL858R	0.001	0.12	0.11	>10	0.25	0.031	0.0078
H1975	EGFRL858R/T790M	0.33	0.3–1	1.2	>10	0.44	0.037	>10
PC-9	EGFR Del19	1–3nM	0.044	0.05	>10	0.13	0.003	0.02
HCC827	EGFR Del19	<0.3nM	0.019	0.063	>10	0.097	0.004	0.0047
H23	EGFR wt	4.4	>10	>10	>10	1.4	4.2	>10
H460	EGFR wt	3.8	4.8	> 10	> 10	2.5	4.0	> 10
A549	EGFR wt	7.5	>10	>10	>10	0.83	5.0	>10
H358	EGFR wt	2.0	>10	>10	>10	4.8	9.4	>10
A431	EGFR wt	0.3–1	1.0	>10	>10	1.2	0.096	0.6
H2122	EGFR wt	1.8	4.5	>10	>10	1.4	7.6	>10

### Ibrutinib displays distinct inhibitory activity against EGFR kinase biochemically and in cell

We then studied the inhibitory activity of ibrutinib against the constructed kinase domain of wt EGFR and mutant EGFR with Promega's ADP-Glo™ assay. The result showed that ibrutinib was most potent against EGFR (L858R/T790M) (IC_50_: 9 nM), moderately potent against EGFR (T790M) (IC_50_: 50 nM), however slightly less potent against wt EGFR (IC_50_: 96 nM). (Table 2 and Fig. S3) A similar activity trend was observed for WZ4002, CO-1686 and AZD9291. (Fig. S3) Mass spectrum study with EGFR(T790M) protein revealed that ibrutinib did inhibit EGFR kinase through formation of a covalent bond with Cys797 amino acid and further confirmed the inhibitory activity loss with PCI-R compound observed in Table 1. (Fig. S4) For auto-phosphorylation of EGFR Y1068 in intact cell lines, ibrutinib exhibited an EC_50_ of 23–58 nM in EGFR primary mutant cell lines (PC-9, HCC827, and H3255) and relatively weaker potency in EGFR L858R/T790M mutant cells (H1975: EC_50_: 145 nM). (Table 3 and Fig. S5) Interestingly, in the wt EGFR-expressing A431 cell line, the auto-phosphorylation of EGFR Y1068 was significantly inhibited by ibrutinib (EC_50_: 49 nM), while WZ4002 (EC_50_: 1436 nM), CO1686 (EC_50_: > 3000 nM) and AZD9291(EC_50_: 818 nM) were much less potent. AZD9291 displayed the strongest inhibitory activities against both the primary (L858R, Del19) and secondary drug resistant mutations (L858R/T790M) EGFR Y1068 phosphorylation, and the best selectivity between the drug resistant mutant and wt (EC_50_: 6 nM versus 818 nM). CO1686 was relatively less potent than AZD9291 and WZ4002, however still exhibited 100-fold selectivity between drug-resistant mutant and wt EGFR Y1068 phosphorylation. These results of inhibitory activities for EGFR Y1068 phosphorylation are in contrast to the results observed in the anti-proliferation assay performed with the A431 cell line (GI_50_: ibrutinib > 10 μM), which suggests that there may be off-targets effects with WZ4002 (GI_50_: 1.0 μM), CO1686 (GI_50_: 1.2 μM) and AZD9291 (GI_50_: 0.096 μM) that contributed to the growth inhibitory activity other than by inhibition of EGFR in A431 cell line which require further more detailed study. (Table 1) In addition, we found that auto-phosphorylation of Y1173 is in general much more sensitive than Y1068 to all of the drugs which is in accordance with the fact the phosphorylation of Y1068 phosphorylation is a prerequisite for phosphorylation of Y1173. [24]

**Table 2:** ADP-Glo biochemical assay of ibrutinib against purified EGFR proteins

Drug/Proteins (IC_50_: nM)	EGFR wt	EGFR (T790M)	EGFR (L858R/T790M)
ibrutinib	96	51	3
WZ4002	44	10	2
CO-1686	114	45	16
AZD9291	134	22	5

**Table 3:** ibrutinib inhibitory efficacy for EGFR Y1068/Y1173 auto-phosphorylation in intact cells

Drug (EC_50_: nM) Y1068/Y1173	H1975	PC-9	HCC827	H3255	A431
ibrutinib	145/63	46/15	58/13	23/20	49/18
WZ4002	7/4	77/19	50/36	42/38	1436/375
CO-1686	29/7	123/10	72/36	250/63	> 3000/1200
AZD9291	6/1	25/24	22/19	17/5	818/108

### Ibrutinib selectively affects mutant EGFR mediated signaling pathways

**Figure 1:**
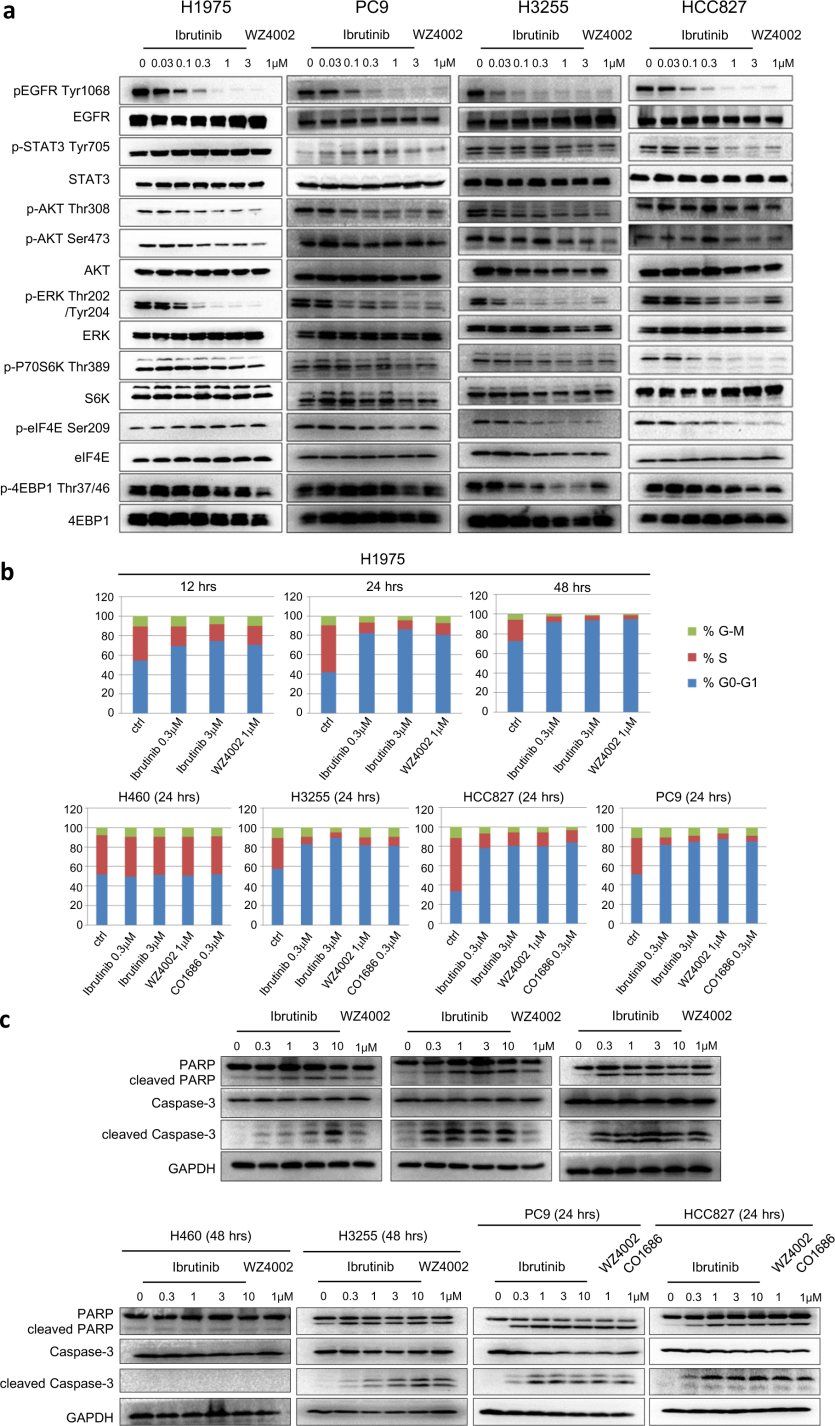
Effect of ibrutinib on EGFR wt/mutant NSCLCs **a.** Ibrutinib effects on wt EGFR and mutant EGFR- mediated signaling pathways; **b.** Ibrutinib selectively arrests mutant EGFR-expressing NSCLC cell lines in G0/G1 phase. **c.** Ibrutinib selectively induces apoptosis in EGFR mutant-expressing NSCLC cell lines.

We then investigated the effect of ibrutinib on EGFR-mediated signaling in both ibrutinib- sensitive and ibrutinib-insensitive NSCLC cell lines. The results demonstrated that ibrutinib potently inhibited both EGFR wt/mutant auto-phosphorylation at Y1068. (Fig. 1A and Fig. S6) In sensitive cell lines (H1975, PC-9, H3255, HCC827) it also potently inhibited targets downstream of ERK. (Fig. 1A and Fig. S7A) Interestingly, it significantly affected the phosphorylation of Akt Thr308 and Ser473 in H1975 cells, however not the other three mutant EGFR-expressing cell lines. (Fig. 1A, Fig. S7B, S7C) Although it did not have apparent enzymatic activity against PI3K kinase. (Fig. S8) This is different from other reported third generation EGFR inhibitors (WZ4002, CO1686 and AZD9291) that can significantly affect the phosphorylation of AktS473 in L858R and del 19 mutant cancer cell lines. In addition, in wt EGFR-expressing NSCLC cell lines, such as H460, A549 and A431, none of the downstream mediators were affected. (Fig. S6)

### Ibrutinb selectively inhibits EGFR mutant cells lines cell cycle progression and induces apoptosis

We next investigated the effect of ibrutinib on cell cycle progression. The FACS results showed that ibrutinib could arrest H1975 cells in G0/G1 phase in a concentration- and time-dependent manner, which mimics the efficacy of WZ4002. (Fig. 1B) A similar effect was observed in PC-9, HCC827 and H3255 cell lines. (Fig. 1B and Fig. S9) However, neither ibrutinib nor WZ4002 or CO-1686 could arrest cell cycle progression in wt EGFR-expressing H460 cells. In addition, ibrutinib also demonstrated a concentration- and time-dependent induction of apoptosis in H1975 cells as evidenced by PARP and Caspase-3 cleavage. This apoptotic effect was also observed in PC-9 and H3255 cell lines, however not in the H460 cell line. (Fig. 1C) These results again validate that ibrutinib is effective against mutant EGFR-expressing cells through induction of cell cycle arrest and apoptosis, however not wt EGFR-expressing cells.

### Ibrutinib slows down EGFR mutant driven NSCLC tumors progression

**Figure 2:**
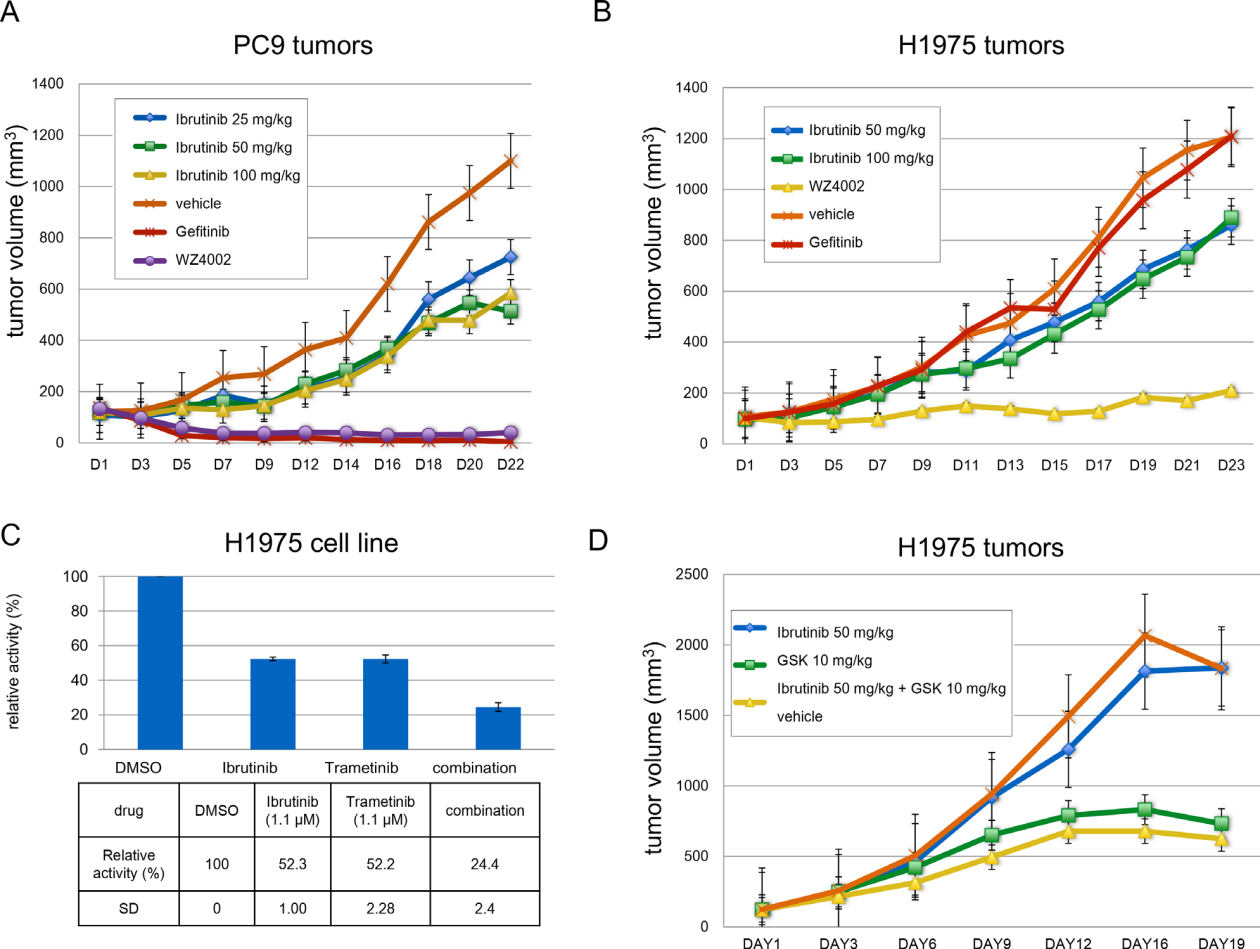
Ibrutinib anti-tumor efficacy in PC-9 and H1975 xenograft mouse models **A.** Ibrutinib slows down tumor progression in a PC-9 xenograft model at 25 mg/Kg, 50 mg/Kg, 100 mg/Kg BID; **B.** Ibrutinb slightly slowed down tumor progression in an H1975 xenograft model at 50 mg/Kg, 100 mg/Kg BID; **C.** Combination study with Ibrutinib and GSK1120212 showed combinatorial anti-proliferation effect against H1975 cells; **D.** Combination of ibrutinib (50 mg/Kg) and GSK1120212(10 mg/Kg) in H1975 xenograft model.

We next determined the anti-tumor activity of ibrutinib in subcutaneous inoculated PC-9 and H1975 xenograft mouse models. In the PC-9 model, continuous ibrutinib treatment for 22 days showed a tumor progression slow down efficacy, though there was no significant difference between the different dosages (25 mg/Kg, versus 50 mg/Kg and 100 mg/Kg BID). (Fig. 2A) However, Gefitinib and WZ4002, which have exhibited similar anti-proliferation effect for PC-9 cells *in vitro*, almost completely suppressed the tumor growth. In the H1975 xenograft mouse model, 23-day continuous (50 mg/kg BID) drug treatment also slowed down tumor progression. Not surprisingly, Gefitinib completely lost the anti-tumor efficacy and WZ4002 almost completely suppressed the tumor growth. Again, a higher dosage of ibrutinib (100 mg/Kg BID) did not improve efficacy. (Fig. 2B)

### Ibrutinib and the MEK inhibitor GSK1120212 combinatorially suppress EGFR (L858R/T790M) mutant H1975 cells proliferation

Considering ibrutinib only exhibited moderate activity against drug acquired resistant mutant EGFR (L858R/T790M)-driven H1975 cells, we sought to explore a drug combination strategy to potentiate the growth inhibitory efficacy of ibrutinib. The EGFR gatekeeper mutant T790M causes drug resistance primarily due to the increased binding affinity to ATP but not the canonical steric hindrance. [25] Several different combination strategies have been explored, such as EGFR monoclonal antibody Cetuximab combination with EGFR inhibitor Afatinib or BIBW2992, allele-specific DNAzyme cDzT with EGFR inhibitor BIBW-2992, and MAPK signaling pathway MEK inhibitor GSK1120212 combination with EGFR inhibitor CO1686. [26–28] Given the fact that both in biochemical assays and cellular colony formation assays ibrutinib and CO-1686 have similar potencies against the EGFR T790M mutation, we then tried to combine MEK kinase inhibitor GSK1120212 with ibrutinib to see if it could potentiate the anti-proliferation efficacy of ibrutinib. The results demonstrated that the combination of Trametinib (used at around the GI_50_ of 1.1μM) with 1.1μM ibrutinib is able to effectively block the growth of the cells. (Fig. 2C and Table S2) However, the combination with ibrutinib (50 mg/Kg, BID) and Trametinib (10 mg/Kg, BID) did not demonstrate apparent combinatorial effect in H1975 tumor xenograft model. (Fig. 2D)

## DISCUSSION

Interestingly, despite the preferred selectivity between wt EGFR and EGFR L858R/T790M shown in the *in vitro* biochemical assays, this trend did not translate into the in-cell auto-phosphorylation assay by looking at Y1068 site. This might be due to assay format differences, since the biochemical assay examines the inhibitory activity against downstream target phosphorylation (here is a peptide substrate), while the auto-phosphorylation assay examines the inhibitory ability to phosphorylate the target protein (EGFR) itself. The other possibility is that in biochemical assay the protein only comprises the kinase domain while in the intact cells the EGFR is full-length. The regulatory domain might play some roles in the protein conformation and activities.

Although ibrutinib exhibited highly potent anti-proliferation efficacy against PC-9 cells, it did not completely suppress the PC-9 cell inoculated tumor progression but only slowed down it. Furthermore, it did not exhibit the dose-dependent efficacy among different dosages in both PC-9 and H1975 cells mediated tumors. However, with the similar formulation, Gefintib and WZ4002 demonstrated much more superior anti-tumor activities against PC-9 and/or H1975 tumor models. One possible reason of this discrepancy between the *in vitro* and *in vivo* might be due to the formulation problems since we did not have access to the clinically used formulation of the drug. However, the other reason might be the unfavorable PK property of ibrutinib against solid tumors since it has been primarily developed against the leukemia (CLL) and lymphoma (MCL) cancers. If, then, an alternative drug formulation, such as nanomaterial-mediated controlled release, might be helpful to improve the anti-tumor efficacy, which requires further detailed study.

In summary, the anti-tumor progression efficacy combined with the already clinically validated safety profile during clinical testing as a BTK kinase inhibitor makes ibrutinib a potentially useful drug candidate for first line treatment of EGFR primary mutation- driven NSCLC. The combination of ibrutinib with other drugs as second line treatment option could also be explored to overcome the EGFR T790M induced drug resistance.

## MATERIALS AND METHODS

### Inhibitors

Ibrutinib, BIBW-2992, W4002, CO-1686, AZD9291, Gefinib and GSK1120212 were purchased from Haoyuan Chemexpress Inc. PCI-R was synthesized in the lab based on the procedure reported previously. [29]

### Cell lines and cell culture

The human cancer cell lines A549, A431, H3255, H1975, PC-9, HCC827, H23, H460, A549, H358 and H2122 cells were purchased from the American Type Culture Collection (ATCC) (Manassas, VA, USA). H1975, PC-9, HCC827, H23, H460, H358, H2122 and EGFR mutant isogenic BaF3 cells lines were cultured in RPMI 1640 media (Corning, USA) with 10% fetal bovine serum (FBS) and supplemented with 2% L-glutamine and 1% penicillin/streptomycin. A549 and A431 were cultured in DMEM media (Corning, USA) with 10% FBS and supplemented with 2% L-glutamine and 1% pen/strep. H3255 was cultured in BEGM media (LONZA, USA) with 10% FBS and supplemented with 2% L-glutamine and 1% pen/strep.

### Antibodies and immunoblotting

The following antibodies were purchased from Cell Signaling Technology (Danvers, MA): Akt (pan) (C67E7) Rabbit mAb (#4691), Phospho-Akt (Ser473) (D9E) XP^®^ Rabbit mAb (#4060), Phospho-Akt (Thr308) (D25E6) XP^®^ Rabbit mAb (#13038), p44/42 MAPK (Erk1/2) (137F5) Rabbit mAb (#4695), Phospho-p44/42 MAPK (Erk1/2) (Thr202/Tyr204) (D13.14.4E) XP^®^ Rabbit mAb (#4370), GAPDH (D16H11) XP^®^ Rabbit mAb, 4E-BP1 (#9644), 4E-BP1 (53H11) Rabbit mAb (#9644), eIF4E (C46H6) Rabbit mAb (#2067), Phospho-eIF4E (Ser209) (#9741), EGF Receptor (D38B1) XP^®^ Rabbit mAb (#4267), Phospho-EGF Receptor (Tyr1068) (D7A5) XP^®^ Rabbit mAb (#3777), Stat3 (#9132), Phospho-Stat3 (Tyr705) (D3A7) XP^®^ Rabbit mAb (#9145), Src (36D10) Rabbit mAb (#2109), Phospho-Src Family (Tyr416) (D49G4) Rabbit mAb (#6943). Antibodies were used at 1:1000.

### EGFR proteins purification for biochemical assay

A construct encoding EGFR residues 696–1022 with a GST tag was cloned into baculovirus expression vector pAcG2T. The protein was expressed by infecting SF9 cells with high titer viral stocks for 48 h. Cells were harvested and lysed in 25 mM Tris (pH 7.9), 150 mM NaCl, and 1 mM DTT. The supernatant was incubated with glutathione Sepharose beads (Genscript). After washing with wash buffer (40 mM Tris pH 7.9, 500 mM NaCl, 1% Glycerol, 1 mM DTT), the beads were incubated overnight with 5ml wash buffer containing 5ul of 5 mg/ml alpha-thrombin to remove GST tag. The eluted EGFR protein was loaded on desalt column PD-10 (GE) to change the buffer to 25 mM Tris pH7.5, 50 mM NaCl, 20 mM MgCl2, and 1 mM DTT. The protein was concentrated to 1 mg/ml and aliquots were frozen and stored at −80C.

### ADP-Glo biochemical assay

The ADP-Glo™ kinase assay (Promega, Madison, WI) was used to screen Ibrutinib, WZ4002, CO1686, and AZD9291 for its EGFR inhibition effects. The kinase reaction system contains 4.95 μl EGFR (WT) (1.5 ng/μl), EGFR (T790M) (6 ng/μl) or EGFR (T790M/L858R) (1.5 ng/μl), 0.55 μl of serially diluted drug, and 5.5 μl EGFR substrate Poly (4:1 Glu, Tyr) peptide (0.2 μg/μl) (Promega, Madison, WI) with 40 μM ATP (Promega, Madison, WI). The reaction in each tube was started immediately by adding ATP and kept going for an hour under 37°C. After the tube cooled for 5 minutes at room temperature, 5 μl solvent reactions were carried out in a 384-well plate. Then 5 μl of ADP-Glo™ reagent was added into each well to stop the reaction and consume the remaining ADP within 40 minutes. At the end, 10 μl of kinase detection reagent was added into the well and incubated for 30 minutes to produce a luminescence signal. Luminescence signal was measured with an automated plate reader (Perkin-Elmer Envision) and each measurement was performed in triplicate.

### Proliferation studies

Cells were grown in 96-well culture plates (2500–3000/well). For adherent cell lines, compounds of various concentrations were added into the plates after cells were cultured for 12 hours. Cell proliferation was determined after treatment with compounds for 72 hours. Cell viability was measured using the CellTiter–Glo assay (Promega, USA), according to the manufacturer's instructions, and luminescence was measured in a multi-label reader (Envision, PerkinElmer, USA). Data were normalized to control groups (DMSO) and represented by the mean of three independent measurements with standard error <20%. GI_50_ values were calculated using Prism 5.0 (GraphPad Software, San Diego, CA).

### Cell cycle analysis

H1975 cells were treated with DMSO, PCI32765 (0.3 μM, 3 μM), WZ4002 (0.3 μM) for 12, 24, 48 hours before cells were harvested by trypsin and washed with cold PBS. HCC827, PC9, H460 and H3255 cells were treated with DMSO, PCI32765 (0.3 μM, 3 μM), WZ4002 (0.3 μM), CO1686 (0.3 μM) for 24 hours before cells were harvested by trypsin and washed with cold PBS. The cells were fixed in 70% cold ethanol and incubated at −20°C overnight then stained with PI/RNase staining buffer (BD Pharmingen). Flow cytometry was performed using a FACS Calibur (BD), and results were analyzed by ModFit software.

### Signaling pathway effect examination

H1975, HCC827, PC9 and H3255 cells were treated with serially diluted PCI32765, WZ4002 (1 μM) for 4 hours. Cells were then washed with PBS and lysed in cell lysis buffer. Phospho-EGF Receptor, Phospho-EGF Receptor (Tyr1068), Stat3, Phospho-Stat3 (Tyr705), AKT, Phospho-AKT (Ser473), Phospho-AKT (Thr308), p44/42 MAPK (Erk1/2), Phospho-p44/42 MAPK (Erk1/2) (Thr202/Tyr204), p70 S6 Kinase, Phospho-p70 S6 Kinase (Thr389) Src, Phospho-Src Family (Tyr416), eIF4E, Phospho-eIF4E (Ser209), 4E-BP1, Phospho-4E-BP1 (Thr37/46) antibody (Cell signaling Technology) were used for immunoblotting.

### Apoptosis effect examination

H1975 cells were treated with serially diluted PCI32765, WZ4002 (1 μM) for 8, 18, 24 hours. H460 cells were treated with serially diluted PCI32765, WZ4002 (1 μM) for 48 hours. PC9, HCC827, H3255 were treated with serially diluted PCI32765, WZ4002 (1 μM) for 24 hours. Cells were then washed in PBS and lysed in cell lysis buffer. PARP, Caspase-3, GAPDH antibody (Cell signaling Technology) were used for immunoblotting.

### Mass spectrum experiment

The purified EGFR protein (30 μg protein in 40 μL buffer of 50 mM Tris, 50 mM NaCl and 20 mM MgCl_2_) was incubated with DMSO or Ibrutinib (50 μM) for 60 min at room temperature. The samples were then subjected to in-solution trypsin digestion. Briefly, the samples were denatured in 8 M urea (160 μl of 10 M urea in PBS was added), reduced by 10 mM of dithiothreitol for 30 min at room temperature (10 μl of 200 mM stock in water was added) and alkylated by 20 mM iodoacetamide for 30 min at room temperature in dark (20 μl of 100 mM stock in water was added). The samples were diluted with ammonium bicarbonate (25 mM, 570 μl) to 2 M urea and subjected to trypsin digestion (Promega; 4 μl of 0.5 μg/μl) overnight at 37°C in the presence of 2 mM CaCl_2_. Digested peptide samples were desalted and re-solubilized in 20uL Buffer A (95% water, 5% acetonitrile, 0.1% formic acid). 2 uL of each sample was analyzed by LC-MS/MS on a Velos Pro Orbitrap Elite mass spectrometer (Thermo Scientific) coupled to an Easy NanoLC. Peptides were eluted from the C18 column using a 30 min gradient of 2–75% Buffer B (100% acetonitrile, 0.1% formic acid). The flow rate through the column was 225 nL/min and the spray voltage was 2.2 kV. The LTQ-Orbitrap was operated in data-dependent scanning mode, with one full MS scan (375–1600 m/z) in the orbitrap followed by MS/MS scans of the 5 most abundant ions using the linear ion trap with dynamic exclusion enabled. The MS data was analyzed by Mascot v2.3.02 using a Uniprot Human sequence database with two differential modifications: Carbamidomethyl modification of 57.02146 on Cysteine and ibrutinib modification of 440.1961 on Cysteine. For the T790M mutant only, an additional “Thr to Met” modification on Threonine was included. For each of the wild-type and mutant samples, the LC-MS/MS analysis was repeated twice with consistent results.

### Colony formation assay

The clonogenic activity of the cells was determined by colony forming assays. Every six conditions of drugs were analyzed (10 μM, 1 μM, 0.1 μM, 0.01 μM, 0.001 μM, DMSO). The cells (H1975, PC-9, HCC827, H3255) were seeded at 600 cells/well in 2 ml medium in 6-well plates. Cells were maintained in a humidified 5% CO_2_ incubator at 37°C for 10 days without changing medium. After 10 days, the assays were stopped and the cells were fixed with 3.5% formaldehyde and 70% ethanol and subsequently stained with 0.02% crystal violet. The total number of colonies exceeding 50 cells per colony was counted and data were given as percentage with respect to the controls.

### Xenograft experiment

Xenograft models. 5 × 10^6^ (PC-9) or 2 × 10^6^ (NCI-H1975) cells (Per 100ul in PBS) were injected subcutaneously into the right flanks of six-week-old female Nude (Nu/Nu) mice (Charles River Laboratories). Animals with 100 mm^3^ tumors were then randomized into groups of 8~10 animals, and dosed twice daily (BID) with Ibrutinib (25, 50, or 100 mg/kg/day) or administrated once daily (QD) with Gefitinib (25 mg/kg/day) or WZ4002 (25 mg/kg/day) by oral gavage for about 3 weeks. Tumor diameter was measured 3 times weekly. Tumor volumes (V) were calculated by the equation (V = length × width × width/2). ALL experiments and procedures followed the guidelines of the Institutional Animal Care and Use Committees of National Institue of Biological Sciences, Beijing.

### Supplemental information

Supplemental Information includes Supplemental Experimental Procedures, nine figures, and two tables and can be found with this article are online available.

## SUPPLEMENTARY MATERIALS


