# Supplementary material for: Ibrutinib selectively and irreversibly targets EGFR (L858R, Del19) mutant but is moderately resistant to EGFR (T790M) mutant NSCLC Cells
Source: Oncotarget. 2015 Sep 5;6(31):31313–22. doi: 10.18632/oncotarget.5182 (PMC4741607; doi:10.18632/oncotarget.5182)
Supplement: Supplementary file 1 [file oncotarget-06-31313-s001.pdf]

SUPPLEMENTARY MATERIALS

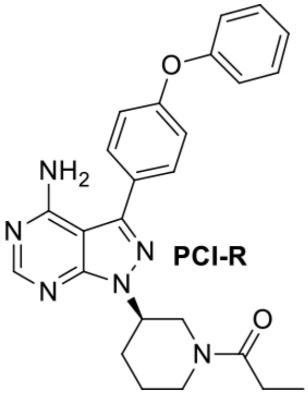

PCI-R

Supplementary Figure S1: Chemical Structure of PCI-R.

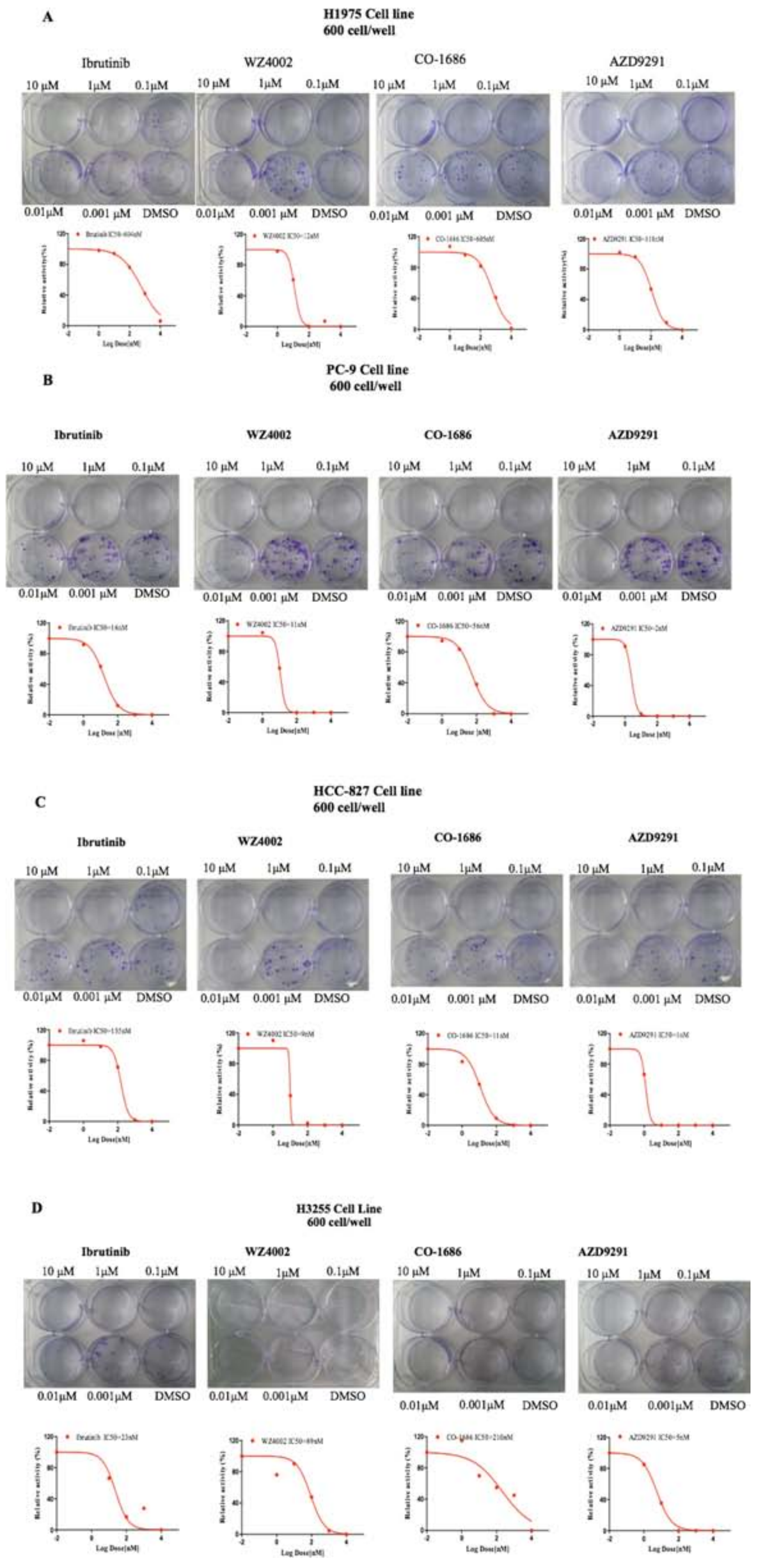

Supplementary Figure S2: Colony formation assay of EGFR inhibitors against EGFR mutant NSCLC cancer cell lines.

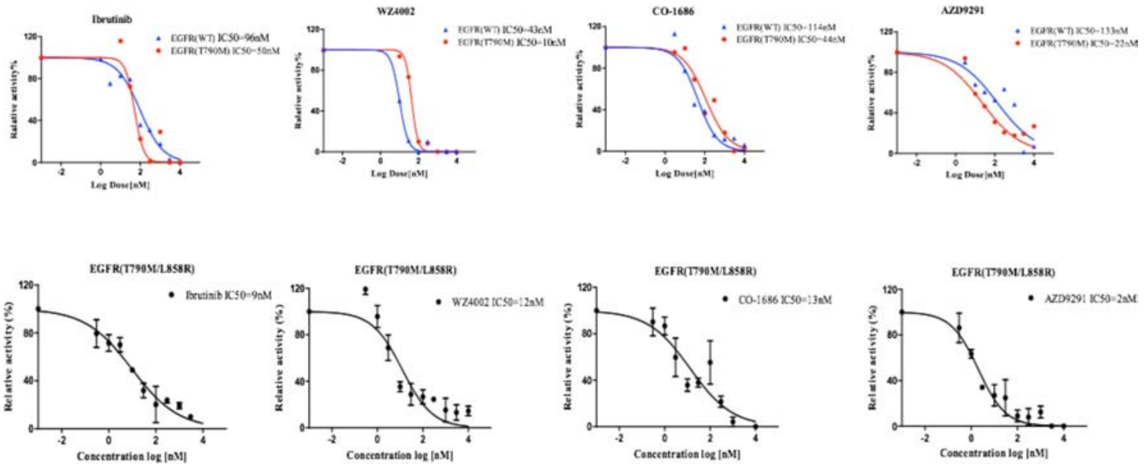

Supplementary Figure S3: ADP-Glo™ biochemical assay of Ibrutinib against EGFRwt, EGFR (T790M) and EGFR (L858R/T790M) proteins.

A.

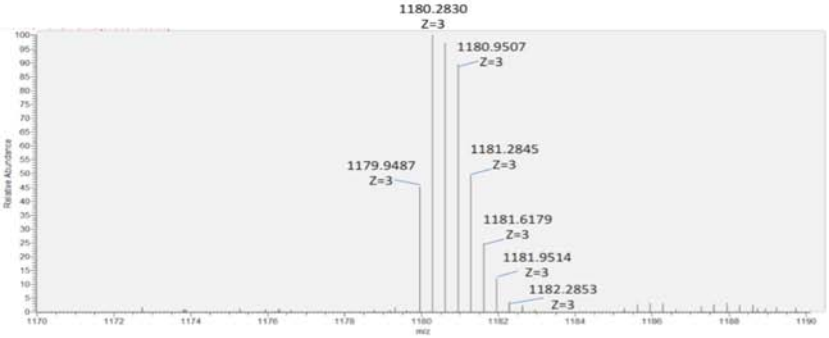

B.

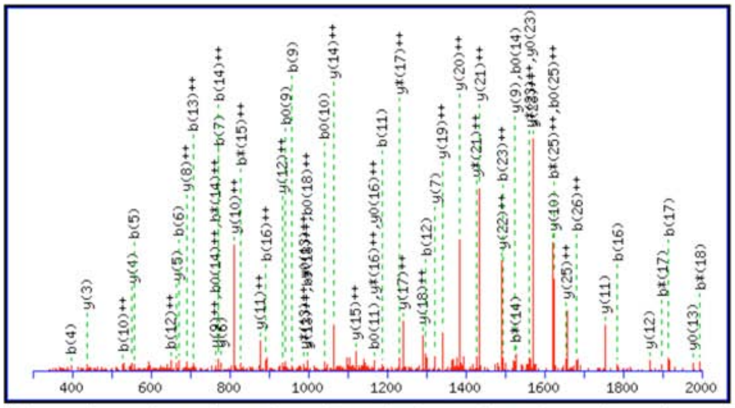

#	b	b++	b*	b*++	b0	b0++	Seq.	y	y++	y*	y*++	y0	y0++	#
1	114.0913	57.5493					L							27
2	227.1754	114.0913					L	3424.7441	1712.8757	3407.7175	1704.3624	3406.7335	1703.8704	26
3	284.1969	142.6021					G	3311.6600	1656.3337	3294.6335	1647.8204	3293.6495	1647.3284	25
4	397.2809	199.1441					I	3254.6386	1627.8229	3237.6120	1619.3096	3236.6280	1618.8176	24
5	557.3116	279.1594					C	3141.5545	1571.2809	3124.5279	1562.7676	3123.5439	1562.2756	23
6	670.3956	335.7015					L	2981.5238	1491.2656	2964.4973	1482.7523	2963.5133	1482.2603	22
7	771.4433	386.2253			753.4328	377.2200	T	2868.4398	1434.7235	2851.4132	1426.2103	2850.4292	1425.7182	21
8	858.4754	429.7413			840.4648	420.7360	S	2767.3921	1384.1997	2750.3656	1375.6864	2749.3815	1375.1944	20
9	959.5230	480.2652			941.5125	471.2599	T	2680.3601	1340.6837	2663.3335	1332.1704	2662.3495	1331.6784	19
10	1058.5914	529.7994			1040.5809	520.7941	V	2579.3124	1290.1598	2562.2858	1281.6466	2561.3018	1281.1546	18
11	1186.6500	593.8286	1169.6235	585.3154	1168.6395	584.8234	Q	2480.2440	1240.6256	2463.2174	1232.1124	2462.2334	1231.6203	17
12	1299.7341	650.3707	1282.7075	641.8574	1281.7235	641.3654	L	2352.1854	1176.5963	2335.1589	1168.0831	2334.1748	1167.5911	16
13	1412.8182	706.9127	1395.7916	698.3994	1394.8076	697.9074	I	2239.1013	1120.0543	2222.0748	1111.5410	2221.0908	1111.0490	15
14	1543.8586	772.4330	1526.8321	763.9197	1525.8481	763.4277	T	2126.0173	1063.5123	2108.9907	1054.9990	2108.0067	1054.5070	14
15	1671.9172	836.4622	1654.8907	827.9490	1653.9066	827.4570	Q	1994.9768	997.9920	1977.9502	989.4788	1976.9662	988.9868	13
16	1785.0013	893.0043	1767.9747	884.4910	1766.9907	883.9990	L	1866.9182	933.9627	1849.8917	925.4495	1848.9077	924.9575	12
17	1916.0418	958.5245	1899.0152	950.0112	1898.0312	949.5192	M	1753.8342	877.4207	1736.8076	868.9074	1735.8236	868.4154	11
18	2013.0945	1007.0509	1996.0680	998.5376	1995.0840	998.0456	P	1622.7937	811.9005	1605.7671	803.3872	1604.7831	802.8952	10
19	2160.1629	1080.5851	2143.1364	1072.0718	2142.1524	1071.5798	F	1525.7409	763.3741	1508.7144	754.8608	1507.7303	754.3688	9
20	2217.1844	1109.0958	2200.1579	1100.5826	2199.1738	1100.0906	G	1378.6725	689.8399	1361.6459	681.3266	1360.6619	680.8346	8
21	2760.3897	1380.6985	2743.3631	1372.1852	2742.3791	1371.6932	C	1321.6510	661.3292	1304.6245	652.8159	1303.6405	652.3239	7
22	2873.4737	1437.2405	2856.4472	1428.7272	2855.4632	1428.2352	L	778.4458	389.7265	761.4192	381.2132	760.4352	380.7212	6
23	2986.5578	1493.7825	2969.5312	1485.2693	2968.5472	1484.7773	L	665.3617	333.1845	648.3352	324.6712	647.3511	324.1792	5
24	3101.5847	1551.2960	3084.5582	1542.7827	3083.5742	1542.2907	D	552.2776	276.6425	535.2511	268.1292	534.2671	267.6372	4
25	3264.6481	1632.8277	3247.6215	1624.3144	3246.6375	1623.8224	Y	437.2507	219.1290	420.2241	210.6157			3
26	3363.7165	1682.3619	3346.6899	1673.8486	3345.7059	1673.3566	V	274.1874	137.5973	257.1608	129.0840			2
27							R	175.1190	88.0631	158.0924	79.5498			1

Supplementary Figure S4: Ibrutinib forms a covalent bond with Cys797 of EGFR as determined by mass spectrometry. A. LC-MS/MS identified a fully tryptic peptide LLGIC(781)LTSTVQLIM(790)QLMPF-GC(797)LLDYVR (m/z = 1179.9490 and z = 3+) with the probe modification on the Cys797 of EGFR-T790M; **B.** MS/MS spectrum and assigned fragment ions of the fully tryptic peptide (m/z = 1180.28 and z = 3+) with the Ibrutinib modification on Cys797 in EGFR-T790M. The MS/MS data were analyzed by Mascot with three differential modifications: Carbamidomethyl modification of 57.02146 on Cysteine, Ibrutinib modification of 440.1961 on Cysteine and “Thr to Met” modification on Threonine. The Mascot ion score of the adduct peptide is 76 and the MS/MS spectrum unambiguously assigned the Ibrutinib modification on Cys797;

(*Continued*)

C.

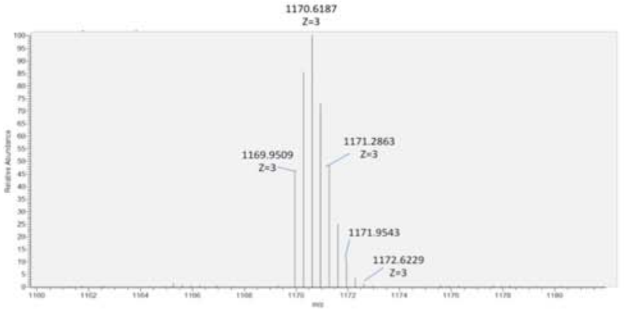

D.

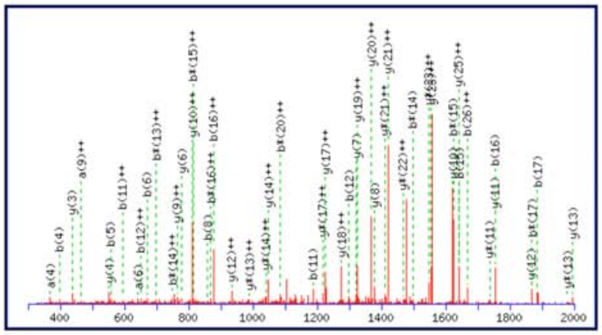

#	a	a++	a*	a*++	b	b++	b*	b*++	Seq.	y	y++	y*	y*++	#
1	86.0964	43.5519			114.0913	57.5493			L					27
2	199.1805	100.0939			227.1754	114.0913			L	3394.7513	1697.8793	3377.7247	1689.3660	26
3	256.2020	128.6046			284.1969	142.6021			G	3281.6672	1641.3372	3264.6407	1632.8240	25
4	369.2860	185.1466			397.2809	199.1441			I	3224.6458	1612.8265	3207.6192	1604.3132	24
5	529.3167	265.1620			557.3116	279.1594			C	3111.5617	1556.2845	3094.5351	1547.7712	23
6	642.4007	321.7040			670.3956	335.7015			L	2951.5310	1476.2692	2934.5045	1467.7559	22
7	743.4484	372.2278			771.4433	386.2253			T	2838.4470	1419.7271	2821.4204	1411.2139	21
8	830.4804	415.7439			858.4754	429.7413			S	2737.3993	1369.2033	2720.3728	1360.6900	20
9	931.5281	466.2677			959.5230	480.2652			T	2650.3673	1325.6873	2633.3407	1317.1740	19
10	1030.5965	515.8019			1058.5914	529.7994			V	2549.3196	1275.1634	2532.2930	1266.6502	18
11	1158.6551	579.8312	1141.6286	571.3179	1186.6500	593.8286	1169.6235	585.3154	Q	2450.2512	1225.6292	2433.2246	1217.1160	17
12	1271.7392	636.3732	1254.7126	627.8599	1299.7341	650.3707	1282.7075	641.8574	L	2322.1926	1161.5999	2305.1661	1153.0867	16
13	1384.8232	692.9153	1367.7967	684.4020	1412.8182	706.9127	1395.7916	698.3994	I	2209.1085	1105.0579	2192.0820	1096.5446	15
14	1485.8709	743.4391	1468.8444	734.9258	1513.8658	757.4366	1496.8393	748.9233	T	2096.0245	1048.5159	2078.9979	1040.0026	14
15	1613.9295	807.4684	1596.9029	798.9551	1641.9244	821.4658	1624.8979	812.9526	Q	1994.9768	997.9920	1977.9502	989.4788	13
16	1727.0136	864.0104	1709.9870	855.4971	1755.0085	878.0079	1737.9819	869.4946	L	1866.9182	933.9627	1849.8917	925.4495	12
17	1858.0540	929.5307	1841.0275	921.0174	1886.0490	943.5281	1869.0224	935.0148	M	1753.8342	877.4207	1736.8076	868.9074	11
18	1955.1068	978.0570	1938.0803	969.5438	1983.1017	992.0545	1966.0752	983.5412	P	1622.7937	811.9005	1605.7671	803.3872	10
19	2102.1752	1051.5912	2085.1487	1043.0780	2130.1701	1065.5887	2113.1436	1057.0754	F	1525.7409	763.3741	1508.7144	754.8608	9
20	2159.1967	1080.1020	2142.1701	1071.5887	2187.1916	1094.0994	2170.1650	1085.5862	G	1378.6725	689.8399	1361.6459	681.3266	8
21	2702.4019	1351.7046	2685.3754	1343.1913	2730.3969	1365.7021	2713.3703	1357.1888	C	1321.6510	661.3292	1304.6245	652.8159	7
22	2815.4860	1408.2466	2798.4595	1399.7334	2843.4809	1422.2441	2826.4544	1413.7308	L	778.4458	389.7265	761.4192	381.2132	6
23	2928.5701	1464.7887	2911.5435	1456.2754	2956.5650	1478.7861	2939.5384	1470.2729	L	665.3617	333.1845	648.3352	324.6712	5
24	3043.5970	1522.3021	3026.5705	1513.7889	3071.5919	1536.2996	3054.5654	1527.7863	D	552.2776	276.6425	535.2511	268.1292	4
25	3206.6603	1603.8338	3189.6338	1595.3205	3234.6553	1617.8313	3217.6287	1609.3180	Y	437.2507	219.1290	420.2241	210.6157	3
26	3305.7288	1653.3680	3288.7022	1644.8547	3333.7237	1667.3655	3316.6971	1658.8522	V	274.1874	137.5973	257.1608	129.0840	2
27									R	175.1190	88.0631	158.0924	79.5498	1

Supplementary Figure S4: (*Continued*). C. LC-MS/MS identified a fully tryptic peptide LLGIC(781)LTSTVQLIT(790) QLMPFGC(797)LLDYVR (m/z = 1169.9512 and z = 3+) with the probe modification on Cys797 of wild-type EGFR. **D.** MS/MS spectrum and assigned fragment ions of the fully tryptic peptide (m/z = 1170.62 and z = 3+) with the Ibrutinib modification on Cys797 in wild-type EGFR. The MS/MS data were analyzed by Mascot with three differential modifications: Carbamidomethyl modification of 57.02146 on Cysteine, Ibrutinib modification of 440.1961 on Cysteine and "Thr to Met" modification on Threonine. The Mascot ion score of the adduct peptide is 51 and the MS/MS spectrum unambiguously assigned the Ibrutinib modification on Cys797.

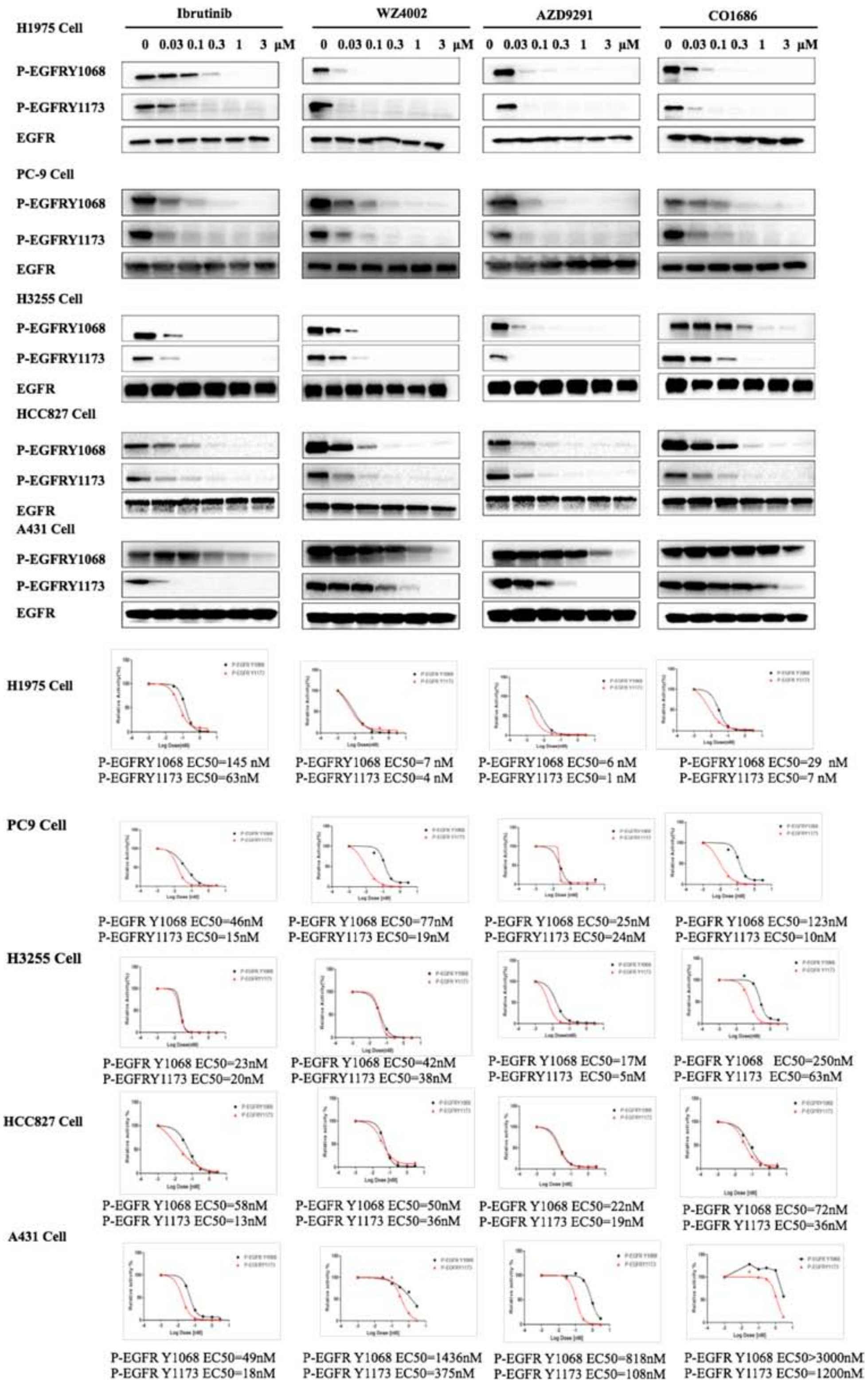

Supplementary Figure S5: Effect of Ibrutinib, WZ4002, AZD9291 and CO1686 on EGFR phosphorylation of tyrosines 1068 and 1173 in EGFR-dependent cancer cell lines.

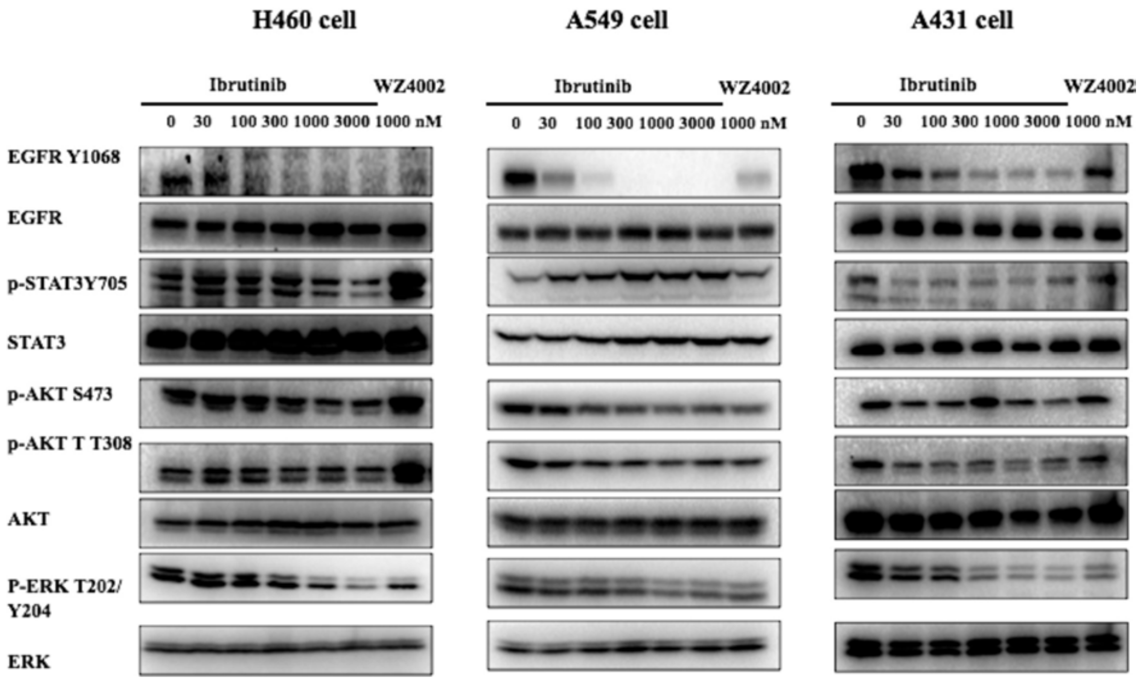

Supplementary Figure S6: Ibrutinib inhibitory effect on EGFR wt mediated signaling pathway.

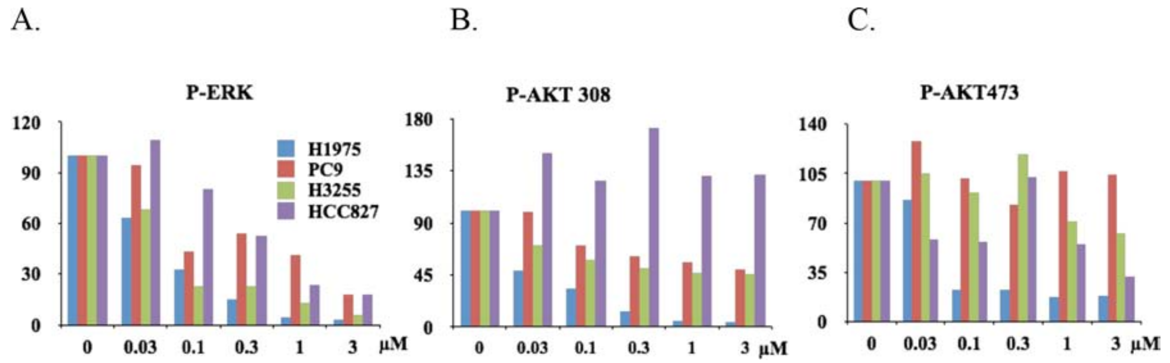

Supplementary Figure S7: Quantification of pERK, pAKT 308/473 and pSrc in drug treated EGFR mutant cell lines.

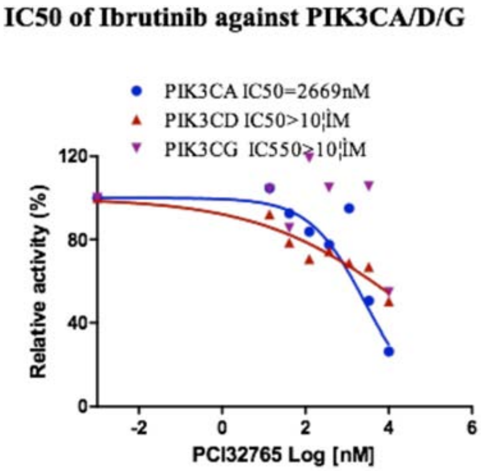

	PIK3CA	PIK3CB	PIK3CD	PIK3CG
PCI32765	2669nM	NA	>10μM	>10μM

Supplementary Figure S8: Ibrutinib inhibitory effect on PI3K kinases by ADP-Glo™ Assay.

A.

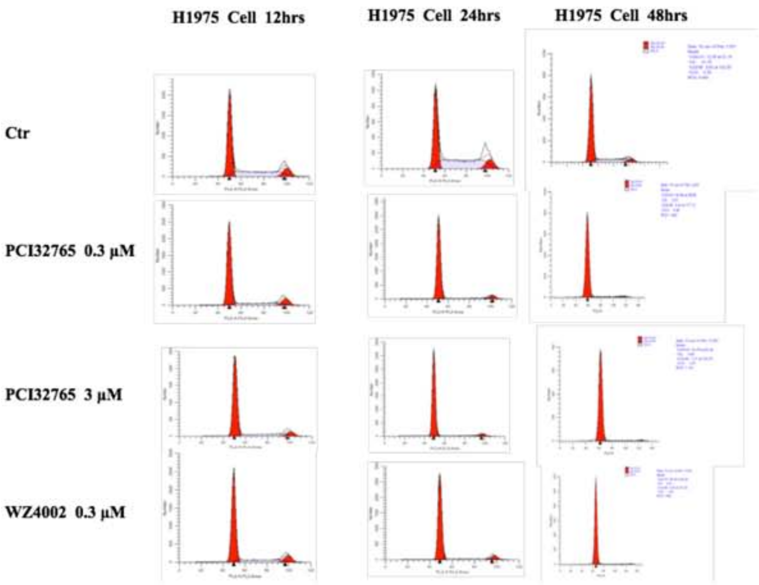

B.

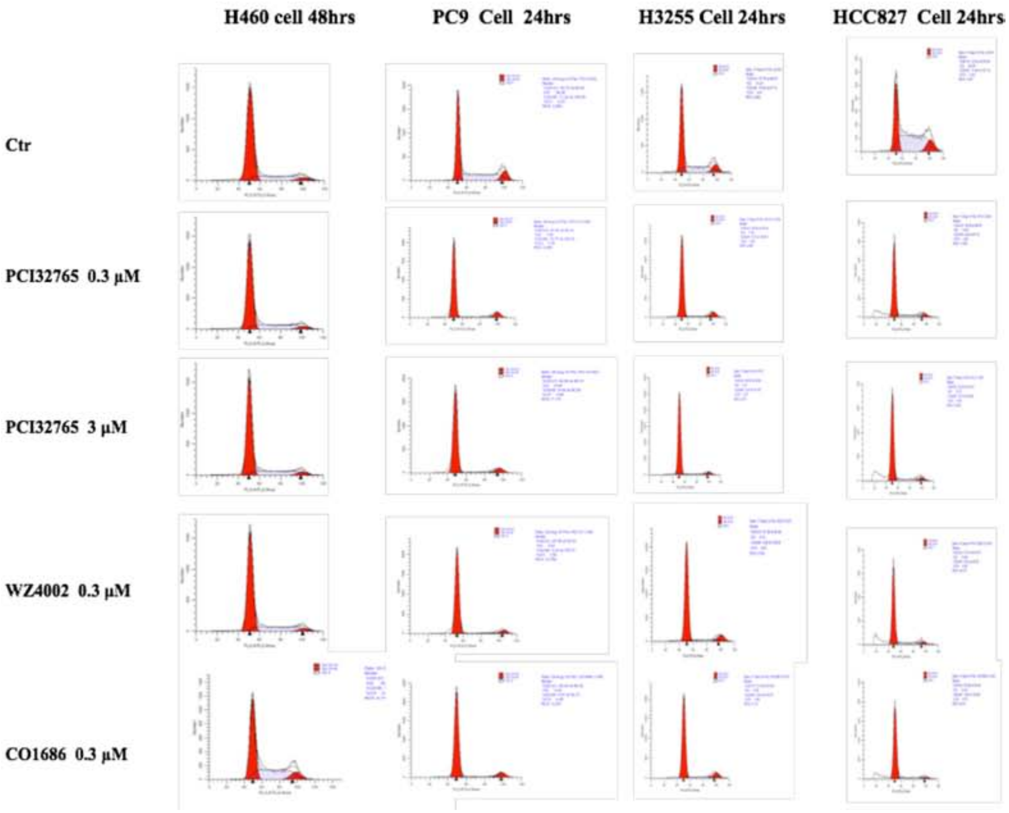

Supplementary Figure S9: Ibrutinib effect on cell cycle progression as determined by fluorescence activated cell sorting (FACS). A. Ibrutinib arrest cell cycle in H1975 cell line **B.** Ibrutinib arrest cell cycle in PC-9, H3255, HCC827 but not H460 cell lines

(*Continued*)

C.

Cell line/Drugs	H1975 Cell line (12h)		H1975 Cell line (24h)		H1975 Cell line (48h)	
Cell line/Drugs	%G0-G1	%G2-M	%G0-G1	%G2-M	%G0-G1	%G2-M
ctr	54.25	10.77	42.2	9.89	72.59	5.63
Ibrutinib 0.3 μM	69.44	10.92	81.96	6.76	92.06	2.43
Ibrutinib 3 μM	74.28	8.07	86.20	4.83	93.79	1.31
WZ4002 0.3 μM	70.4	10.17	80.35	7.63	94.64	0.95

D.

Cell line/Drugs	H460 Cell Line (48h)		PC-9 Cell Line (24h)		H3255 Cell Line (24h)		HCC827 Cell Line (24 h)	
% cell cycle stage	%G0-G1	%G2-M	%G0-G1	%G2-M	%G0-G1	%G2-M	%G0-G1	%G2-M
ctr	72.64	4.67	50.73	11.22	57.79	10.98	33.53	11.46
Ibrutinib0.3 μM	69.88	5.02	81.91	10.77	82.82	9.73	78.26	6.86
Ibrutinib 3 μM	70.49	5.09	84.9	8.54	89.05	5.24	80.37	5.91
WZ4002 0.3 μM	71.39	5.15	87.95	6.24	81.96	9.90	79.74	5.62
CO1686 0.3 μM	51.62	8.78	85.49	8.97	81.24	9.84	83.96	3.62

Supplementary Figure S9: (*Continued*). C. Quantification of Ibrutinib arrest cell cycle in H1975 cell line **D.** Quantification of Ibrutinib arrest cycle in PC-9, H3255, HCC927 and H460 cell line

Supplementary Table S1: Effect of ibrutinib oncolony formation inEGFR mutant NSCLC cancer cell lines.

Drug/Cell lines (EC_{50}: nM)	H1975 EGFR (L858R/ T790M)	PC-9 EGFR del 19	HCC827 EGFR Del 19	H3255 EGFR L858R
ibrutinib	604.7	16.14	155.3	23.40
WZ4002	12.18	11.11	9.649	89.06
CO-1686	605.8	56.25	11.51	210.8
AZD9291	118.1	2.507	1.234	5.592

Supplementary Table S2: Ibrutinib combined with Trametinib against H1975 cell lines growth.

		Trametinib									
		DMSO	10 µM	3.3 µM	1.1 µM	0.37 µM	0.12 µM	0.04 µM	0.014 µM	0.0045 µM	0.0015 µM
PCI-32765		100	20.59	66.83	77.20	80.79	78.86	79.50	77.33	77.24	82.21
	3.3 µM	24.22	3.65	13.55	20.98	20.73	22.68	21.58	21.23	18.24	18.13
	1.1 µM	34.56	6.99	18.20	26.69	28.00	30.35	31.81	27.27	24.20	24.73
	0.37 µM	43.77	13.98	27.96	37.80	43.93	41.62	41.01	35.86	33.13	33.73
	0.12 µM	77.65	16.76	43.52	56.41	57.51	59.86	55.01	47.42	49.09	50.42
	0.040 µM	103.98	39.99	61.08	66.36	66.06	66.37	61.66	63.65	64.53	64.97
